# Attention and normalization circuits in macaque V1

**DOI:** 10.1111/ejn.12857

**Published:** 2015-03-11

**Authors:** M Sanayei, J L Herrero, C Distler, A Thiele

**Affiliations:** 1 Institute of Neuroscience, Newcastle UniversityNewcastle upon Tyne, NE2 4HH, UK; 2 Allgemeine Zoologie und Neurobiologie, Ruhr-Universitaet BochumBochum, Germany

**Keywords:** attention, normalization, orientation tuning, striate cortex, surround suppression

## Abstract

Attention affects neuronal processing and improves behavioural performance. In extrastriate visual cortex these effects have been explained by normalization models, which assume that attention influences the circuit that mediates surround suppression. While normalization models have been able to explain attentional effects, their validity has rarely been tested against alternative models. Here we investigate how attention and surround/mask stimuli affect neuronal firing rates and orientation tuning in macaque V1. Surround/mask stimuli provide an estimate to what extent V1 neurons are affected by normalization, which was compared against effects of spatial top down attention. For some attention/surround effect comparisons, the strength of attentional modulation was correlated with the strength of surround modulation, suggesting that attention and surround/mask stimulation (i.e. normalization) might use a common mechanism. To explore this in detail, we fitted multiplicative and additive models of attention to our data. In one class of models, attention contributed to normalization mechanisms, whereas in a different class of models it did not. Model selection based on Akaike's and on Bayesian information criteria demonstrated that in most cells the effects of attention were best described by models where attention did not contribute to normalization mechanisms. This demonstrates that attentional influences on neuronal responses in primary visual cortex often bypass normalization mechanisms.

## Introduction

Attention aids perceptual abilities. The neuronal underpinnings of this have been studied in detail, whereby some studies have reported that attention changes tuning curves, whereas others have argued that it changes neuronal gain in either a multiplicative manner, an additive manner or that it mostly affects contrast gain (Spitzer *et al*., 1988; McAdams & Maunsell, 1999; Reynolds *et al*., 1999, 2000; Treue & Maunsell, 1999; Martinez-Trujillo & Treue, 2004; Williford & Maunsell, 2006; Roberts *et al*., 2007; Thiele *et al*., 2009). Normalization models of attention can reconcile many of these apparently conflicting results (Ghose, 2009; Lee & Maunsell, 2009; Reynolds & Heeger, 2009), with notable success in explaining the interactions between attention and stimulus contrast (Reynolds & Heeger, 2009; Herrmann *et al*., 2010). Normalization can be viewed as a canonical neural computation (Carandini & Heeger, 2012), which computes the ratio between the activity of a neuron and the summed activity of a large pool of neurons. Normalization is thus a divisive (non-linear) operation, and it has been successfully used to explain properties of neurons at different stages of sensory processing, in different model organisms and zoological phyla, as well as different cognitive operations (for a review see Carandini & Heeger, 2012). It ensures that neurons retain a sufficient dynamic range under varying input regimes, and thereby preserves selectivity of responses. One of the phenomena captured by divisive normalization is centre surround suppression (Cavanaugh *et al*., 2002a). As pointed out above, normalization models have been used also to explain certain effects of attention on neuronal activity in cortical areas. Normalization models argue that attention affects neuronal processing by interacting multiplicatively with the stimulus drive and with the suppressive drive (Reynolds & Heeger, 2009). As a consequence, attention itself contributes to normalization circuits, and response normalization is not only determined by the stimulus size, but also by the strength of attention and the size of the attentional field. Normalization should therefore affect different parameters of neuronal tuning functions in a manner similar to attention. This proposal cannot strictly be true, as normalization usually results in a reduction of neuronal activity (Carandini *et al*., 1997; Britten & Heuer, 1999; Anderson *et al*., 2000; Sceniak *et al*., 2001; Albrecht *et al*., 2002; Cavanaugh *et al*., 2002a; Carandini & Heeger, 2012), while attention most often results in an increase (Moran & Desimone, 1985; Luck *et al*., 1997; Roelfsema *et al*., 1998; Ito & Gilbert, 1999; McAdams & Maunsell, 1999; Thiele *et al*., 1999; Treue, 2001; Ghose & Maunsell, 2002; Martinez-Trujillo & Treue, 2002; Reynolds & Chelazzi, 2004; Williford & Maunsell, 2006; Mitchell *et al*., 2007; Roberts *et al*., 2007). However, across a neuronal population the effects of stimulus-driven and attention-driven normalization could still be correlated, even if the average sign of the effect differs between the two. For example, only few neurons show increased responses upon presentation of stimuli that strongly engage normalization mechanisms, such as surround/mask stimuli (Walker *et al*., 2000). However, those that do might show reduced firing rates induced by attention. Conversely, neurons most strongly suppressed by surround stimuli might show the largest activity increase when attention is deployed to the neuron's receptive field (RF). It suggests that surround/mask stimulus modulation indices would be correlated with attention modulation indices. This has been demonstrated for tuned normalization of middle temporal area (MT) neurons (Ni *et al*., 2012), where normalization was tested when two stimuli were in the RF. Under those conditions a normalization model yielded a good description of the data. A similar study performed in area V4, however, came to the conclusion that attention acts most likely by altering the input gain of neurons (Ghose & Maunsell, 2008), while it acts through normalization mechanisms in < 40% of the neurons. To what extent normalization models also account for attentional effects in primary visual cortex is currently unknown. Moreover, it is unclear to what extent alternative models, not employing normalization mechanisms, yield equivalent or better fits. In area V1 the effects of attention cannot be compared when attention is alternately directed to one of two stimuli, both placed inside the RF, because V1 (parafoveal) RFs are too small for such a manipulation. We thus compared the effects of attention (inside the RF vs. outside the RF) and of surround/mask stimuli on V1 orientation tuning functions and on V1 firing rates. We used Akaike's and Bayesian information criteria for model selection and found that for the majority of V1 cells attention is best described by models where attention does not contribute to normalization.

## Methods

All procedures were carried out in accordance with the European Communities Council Directive RL 2010/63/EC, the US National Institutes of Health Guidelines for the Care and Use of Animals for Experimental Procedures, and the UK Animals Scientific Procedures Act. The study received institutional review board committee approval (Animal Welfare and Ethical Review Body – AWERB, Newcastle University). In the present investigation two adult awake male macaques (*Macaca mulatta*, age 7–9 years, weight 11–13 kg) were used.

### Surgical preparation

The monkeys were implanted with a headpost and recording chambers over area V1 under sterile conditions and under general anaesthesia. Surgery and post-operative care were identical to that published in detail previously (Thiele *et al*., 2006).

At the end of the experiments the animals were killed with an overdose of pentobarbital and perfused through the heart. Details of the perfusion and histological procedures are given in Distler & Hoffmann (2001). The location of the recording sites in area V1 was verified in histological sections stained for cyto- and myeloarchitecture.

### RF mapping

The location and size of RFs was measured by a reverse correlation method. A black square (0.1° size, 100% contrast) was presented at pseudorandom locations on a 1° by 1° sized grid, spaced into 10 × 10 squares of 0.1° (five repetitions for each location, 100-ms stimulus presentation, 100-ms interstimulus interval), while monkeys kept fixation on a central fixation point. Details of the RF mapping were published previously (Gieselmann & Thiele, 2008). RF eccentricity in this study ranged from 2° to 5°, and RFs were located in the lower quadrants of the visual field.

### Behavioural task and stimuli

**Figure 1:**
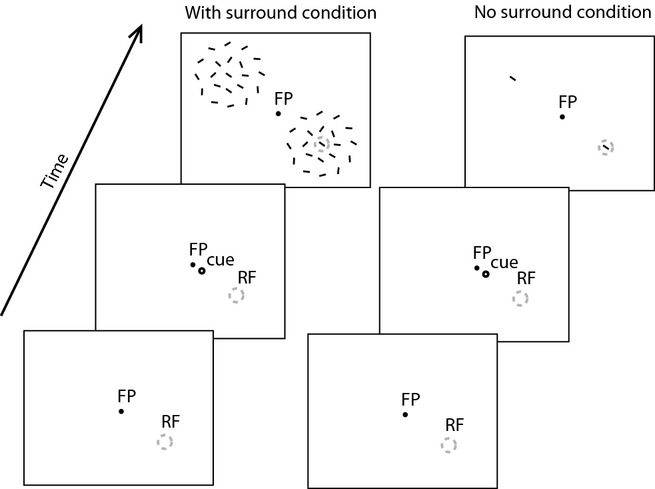
Task and stimuli used. Animals were required to fixate a fixation point (FP) on the monitor. A cue indicated to the animal where to attend to on the current trial. Then, 900 ms after cue offset two stimuli appeared, one centred on the neuron's RF and one in the opposite hemifield equidistant to the FP. Animals had to detect a subtle change in the middle of the centre bar in the cued location, and ignore changes in the un-cued location. The left timeline shows the conditions when the centre stimulus was surrounded by distractor stimuli (with surround condition). The right timeline shows the condition when the centre bar was presented in isolation (no surround condition). The exact orientation of surrounding bars used on every trial in the experiments is shown here.

Monkeys were trained to fixate a red fixation point (FP, 0.1° diameter) on a grey background (38 cd/m^2^) presented centrally on a 20-inch analogue cathode ray tube monitor (110 Hz, 1600 × 1200 pixels, 57 cm from the animal). Eye position was monitored with an infrared-based system (220 Hz, Thomas Recording, Giessen, Germany) with a fixation window of ±0.7–1.1°. Each trial was initiated when the monkey held the touch bar and fixated the central point (Fig.1). Then, 400 ms after fixation onset, a cue (blue annulus, 0.24° outer diameter, 0.18° inner diameter) was presented for 400 ms. The cue indicated to the monkey the location it had to attend to. The location to be attended to was spatially offset from the cue location. The cue appeared at one-quarter the distance between RF centre and fixation point, or at the equivalent location in the opposite visual hemifield on ‘attend away’ trials. The location the animal had to attend to was either at the centre of the RF or at an equivalent location in the opposite visual hemifield (on attend away trials). Following a 900-ms blank period, ensuring temporal separation between cue offset and stimulus onset, two identical bars appeared, one in the neuron's RF and one in the opposite hemifield. The spatial and temporal offset between cue and stimulus location was introduced to minimize effects of, for example, adaptation/summation/facilitation at the neuron's RF. The difference in cueing location and RF location could in principle have resulted in some spatial uncertainty about the allocation of focus of attention. However, we believe that this will have been a small effect, as the animals would, on a daily basis, rapidly know where the stimuli would appear and thus where the focus of attention should be directed to. Moreover, any spatial uncertainty would be eliminated with the onset of the bar stimuli. Stimuli were dark bars (0.5° length and 0.1° width, 12.5 cd/m^2^). Stimulus orientation varied pseudo-randomly from trial to trial in steps of 15°, ranging from 0 to 165° (i.e. 12 different orientations). After 500–800 ms (randomly assigned in steps of 1 ms) a brighter patch (0.1°× 0.1°, 28 cd/m^2^) appeared at the centre of one of the bars. The patch had 50% probability of being at the cued (target) or un-cued (distracter) location. If the patch occurred in the cued location monkeys had to release the touch bar within 500 ms to receive a juice reward. If the patch occurred in the un-cued location, the monkeys had to wait for the second patch to appear at the cued location. The bars were presented either alone or centred on the RF of the neuron (and at an equivalent distance in the opposite hemifield), or the centre bars were surrounded by multiple orientated bars. The surround bars were identical in size and luminance to the bar at the centre, but varied in orientation. The surround bars were placed equidistantly on two concentric circles (circle radius 1° and 2°, eight bars on the inner circle and 12 bars on the outer circle) around the centre bar location (Fig.1). The distribution of surround orientations was the same for all the experiments reported here, and it was identical to the orientations shown in Fig.1. The distance between individual surround bars was 0.8° for the inner circle and 1° for the outer circle, respectively (centre-to-centre). To determine the influence of the surround bars on neuronal responses in the absence of the central bar stimulation we also presented the surround bars in isolation. We refer to this as the ‘surround only’ condition. The monkey's task in the ‘surround only’ condition was to detect the occurrence of the patch at the cued location, but on those trials the patch occurred on the background, rather than on the bar. Its location in the visual field was unaffected by the presence or absence of a central bar.

The surround/mask stimuli activate normalization mechanisms (Carandini & Heeger, 2012), and they therefore yielded for every cell an estimate of to what extent neuronal activity is affected by surround normalization. This effect could then be compared with the effect of attention on neuronal firing, and thereby assess whether attention employs normalization mechanisms (see Data analysis below). Note that we do not make a specific distinction between surround and mask stimuli here, as the surround may have intruded on some occasions into the neuron's summation area. Based on minimal RF mapping the surround bars were presented outside the classic RF, but some surround/mask stimuli presented on their own nevertheless elicited a residual response in some of our neurons (for details see below under Surround intrusion).

The order of stimulus and attend conditions was presented in pseudorandom manner. Neither attention nor surround conditions were blocked.

### Data analysis

We analysed neuronal responses for the different stimulus conditions in a response window from 200 to 500 ms after stimulus onset. This period captured the sustained response where attentional modulation is usually more profound (Roelfsema *et al*., 1998; Roberts *et al*., 2007), although this may depend on the task (Ghose & Maunsell, 2002). Within this response period we calculated the stimulus-driven activity for the different centre stimulus orientations, for the conditions when no surround stimulus was present [referred to as *R*(*C*), whereby *R* indicates ‘response’ and *C* indicates ‘RF centre’], as well as the conditions when a surround stimulus was present [referred to as *R*(*C*,*M*), whereby *M* represents ‘mask/surround’]. This was done separately for the attend RF [*R*(*C*_att_) and *R*(*C*_att_,*M*)] and for the attend away [*R*(*C*) and *R*(*C*,*M*)] conditions. Additionally we determined the response in the ‘surround only’ condition for attend RF [*R*(*M*_att_)] and for attend away trials [*R*(*M*)].

To determine the orientation tuning at the population level for the different stimulus and behavioural conditions, we normalized the activity of each cell by dividing all stimulus-driven responses by the response obtained when the preferred orientation in the ‘attend RF –no surround’ [*R*(*C*_att_)] condition was presented. To establish the time course of attentional modulation for the different stimulus conditions we calculated the population peri-stimulus-time histogram (PSTH) for the population of cells when the preferred orientation was present. This was done based on normalized single-cell activity, whereby the maximum of the response in the attend RF – no surround condition [*R*(*C*_att_)] was taken as the denominator in the normalization process.

#### Surround intrusion

For each cell we determined whether the ‘surround only’ stimulus affected neuronal activity by comparing the surround-driven response (if any) [*R*(*M*)] with the spontaneous activity (300 to 0 ms before stimulus onset) and determined whether the two differed significantly (*t*-test). Cells where the ‘surround only’ stimulus induced significant responses were labelled as such and could be excluded from analysis as necessary, to control for surround intrusion effects (for details see Results section and explanation therein). As an additional control we subtracted the response during the ‘surround only’ condition response from the responses when the surround and the centre stimulus was present. For those controls we then also subtracted the spontaneous activity from the centre only responses (i.e. when no surround stimuli were presented) to control for offset effects.

### Orientation tuning

A wrapped Gaussian function was fitted to the mean response elicited by each centre bar orientation in the four different conditions (least square fitting):


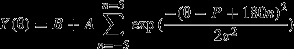


Here *Y*(θ) is the predicted response for the given bar orientation (θ), *B* is the baseline, *A* is the amplitude of the tuning curve, *P* is the preferred orientation and σ is the bandwidth of the tuning curve. We assessed the goodness of each fit by calculating the χ^2^ error between the data and the model predictions (Press *et al*., 2002). The fitting was performed such that the baseline value was within 20% of the minimum response obtained for the respective attention/surround condition, and the sum of the baseline plus amplitude was within 20% of the maximum response obtained for the respective attention/surround condition. This was done to ensure a fit which describes all aspects of the data well, and does not perform too much of a trade-off between, for example, the bandwidth and the baseline vs. the amplitude parameter. Otherwise the fitting might result in parameter estimates where, for example, the amplitude reaches very large (or small) values, which would not describe the maximal measured response, while the overall fit could still be good. Based on this fitting procedure we calculated the percentage of variance accounted for by the model (Carandini *et al*., 1997), and included cells into further analysis of tuning parameter changes, provided the fits for the four conditions (attend away/attend RF with and without surround) accounted for ≥50% of the variance. Based on these fits we determined the orientation index, which was defined as OI = *B*/(*A*+*B*). We excluded cells where the OI was < 0.33. As an additional control we excluded cells where the maximum response did not exceed the minimum response by at least 5 spikes/s.

To determine the effect of surround stimuli on the above listed estimates we calculated a *surround modulation* index (MI_surr_) according to:


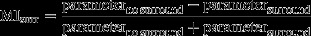


where *parameter* was either the amplitude, baseline or tuning width (calculated as half width at half height of the tuning curve, HWHH). This yielded separate MI_surr_ values for the attend away condition and for the attend RF condition. We also calculated the *attention modulation* index (MI_att_) according to:


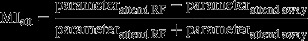


Again, *parameter* represented either the amplitude, baseline or tuning width. Thus, the parameters yielded separate MI_att_ values for the no surround and the surround conditions.

#### Modulation indices based on firing rates

In addition to calculating MIs based on the orientation tuning curve parameters, we also calculated MIs based on raw firing rates, using the above formulas, but replacing ‘parameter’ with ‘firing rate’. This was done for preferred orientation firing rates, and for firing rates averaged across all stimulus orientation conditions.

#### Strength of surround suppression

To determine the strength of surround suppression based on the measured, not fitted data, we calculated the suppression index (SI) as


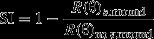


where *R*(θ)_no surround_ was the response to the preferred orientation in the attend away condition when no surround was present, and *R*(θ)_surround_ was the respective response in the condition when a surround was present (attend away). The preferred orientation was taken to be the stimulus orientation that was closest to the location of the maximum of the fitted wrapped Gaussian function.

### Correlations between surround suppression strength and attentional modulation

To determine whether surround stimulus-induced changes in a tuning parameter (or in raw firing rates) were correlated with changes in any of the other parameters (raw firing rates), we calculated Spearman rank correlations using the respective MIs. To further determine whether attentional MIs were correlated with surround MIs, we calculated Spearman rank correlations using the attentional MIs obtained with and without surround and correlated it with the surround MI obtained from the attend away condition. We also calculated Spearman rank correlations between attentional MIs and surround MIs in the attend RF conditions.

### Model fitting

Normalization captures the phenomenon of surround suppression and the phenomenon of masking. The response of a V1 neuron to an orientated bar of a given contrast is suppressed by the presence of various orientated bars presented either at the fringe or outside the neuron's summation area. Within the context of this study, we emphasize the notion of ‘fringe or outside the neuron's summation area’, as the surround stimuli presented alone elicited a small response in some of our neurons. For the purpose of this paper the difference between pure surround suppression and some form of masking (i.e. non-preferred stimuli placed in the summation area) is not very important, as the equations that describe the two forms of normalization are basically identical (Carandini & Heeger, 2012). Specifically, the phenomenon of surround and mask suppression can be captured by the following formula:


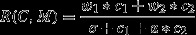
(1)

where *R*(*C*,*M*) denotes the response of the neuron to a given centre orientation when the surround/mask stimulus is simultaneously present. *w*_1_ corresponds to the drive the neuron receives given the orientation of the centre stimulus, *c*_1_ is the contrast of the centre stimulus, and *w*_2_ and *c*_2_ are the respective drives and contrasts for the surround/mask stimulus. As different neurons show different amounts of normalization, a normalization scaling term α is added to the equation. The symbol σ determines how the response saturates with increasing contrast, and also prevents division by zero. In our study we did not determine the contrast response function of neurons. We thus did not know the value σ for each neuron, but rather fixed it to be 0.2. We also used different values, and we allowed it to be a free parameter in the fitting procedures (see below), but none of these manipulations changed the main conclusions, and we thus report results obtained when σ = 0.2. Values of *c*_1_ and *c*_2_ were either 0.32 or 0. We redefined them to be 1 or 0, i.e. stimulus on or stimulus off. This was done because we used the response measured at 32% contrast for the fitting (see below), and this response should thus not be scaled by the contrast itself. In the absence of attention, *w*_1_ then corresponds to the neuron's response to a given centre orientation, and *w*_2_ corresponds to the neuron's response to the surround/mask only stimulus (i.e. when no centre stimulus was present and attention was directed to the opposite hemifield).

#### Multiplicative attention models

When attention is directed to the RF, the response to the centre and mask/surround stimulus *R*(*C*_att_,*M*_att_) can be predicted from the attend away responses by altering Eq. (1) as follows:


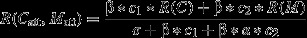
(2)

where *R*(*C*) and *R*(*M*) were the respective responses to the centre and to the mask stimulus when presented in isolation (after subtraction of spontaneous activity), β corresponds to the attention term and α corresponds to the surround/mask normalization scaling term. Equation (2) assumes a large attention field as β affects centre and surround/mask responses. A small attention field would be captured by


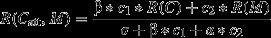
(3)

where β only affects centre responses.

Equations (2) and (3) can also be used to predict *R*(*C*_att_) from *R*(*C*) responses, i.e. the response to an attended centre only stimulus from unattended centre only stimulus, by setting *c*_2_ = 0. Similarly, *R*(*C*,*M*) (i.e. the unattended response to a centre and surround stimulus) can be predicted from the centre only and surround only unattended responses, by setting β = 1. Equally, *R*(*C*_att_, *M*_att_) responses can be predicted from *R*(*C*_att_) and *R*(*M*_att_) responses, by setting β = 1.

Ni *et al*. (2012), argued for a model where attention and mask stimuli equally contribute to normalization, and that differences in normalization strength were sufficient to explain differences in attentional modulation at the firing rate level. To account for this, they used a single fixed attentional parameter β for their data fitting. This was implemented in our case by forcing β to be fixed when attention was directed to the RF and β = 1 otherwise. The fixed β values represented the population mean attentional modulation (mean β) that was obtained for the multiplicative and additive model, respectively, when β was allowed to vary freely (multiplicative model mean β = 1.30, additive model mean β = 7.39).

To compare normalization models of attention with models where attention does not contribute to normalization we changed Eqns (2) and (3) such that the attention term β does not appear in the denominator. This is shown in Eqns (4) and (5):


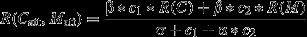
(4)


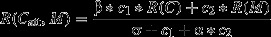
(5)

These models would thus argue for simple multiplicative scaling of responses by attention.

#### Additive attention models

In V1 the effects of attention were sometimes best accounted for by additive attention models (Buracas & Boynton, 2007; Thiele *et al*., 2009). To test the validity of these models in the context of this study we altered Eqns (2)–(5) such that the attention term would be additive, rather than multiplicative:


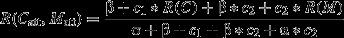
(6)


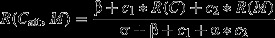
(7)

For the case where attention was directed away from the RF, β was set to 0 [we also tested models where β was set to 1 as in the multiplicative models (even if we felt it was not sensible to do so), which gave qualitatively similar results to those reported below]. The single parameter additive model (as described above for the multiplicative case) was tested by setting β = 7.39 (mean population β obtained for the additive model when β was allowed to vary freely), for conditions when attention was directed to the RF, and β = 0 (or 1 as described above) otherwise.

Finally, an additive attention model, where attention does not contribute to normalization was implemented as:


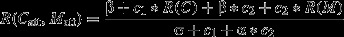
(8)


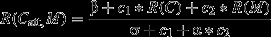
(9)

Note, that in Eqns (6) and (8), β is still multiplied by c_2_, as it should not affect the second half of the numerator for conditions when the surround stimulus had 0% contrast. The effect under those conditions would still be additive as the product (β * c_2_) is added to the R(M) response, rather than multiplying it.

We used unconstrained non-linear optimization to determine the parameters α, and β (χ^2^ error minimization) aiming to predict the following responses:

1. Effect of normalization: *R*(*C*,*M*) from *R*(*C*) and *R*(*M*) responses (i.e. the surround/mask normalization in the absence of attention from centre only responses and mask only responses) for each of the 12 different centre orientations [here β was set to 1 for the multiplicative models and 0 (but see above) for additive models].
2. Effect of normalization: *R*(*C*_att_,*M*_att_) from *R*(*C*_att_) and *R*(*M*_att_) responses, i.e. another determination of normalization effects for each of the 12 different centre orientations [β was set to 1 for the multiplicative models and 0 (but see above) for additive models for this condition again].
3. Effect of attention: *R*(*C*_att_) from *R*(*C*) responses (i.e. the effect of attention on centre only responses from centre only responses (attend away condition) for each of the 12 different centre orientations.
4. Effect of attention: *R*(*C*_att_,*M*_att_) from *R*(*C*) and *R*(*M*) responses (effects of attention on centre and surround/mask responses predicted from attend away – no surround and from attend away surround only responses) for each of the 12 different centre orientations.
5. Effect of attention: *R*(*C*_att_,*M*_att_) from *R*(*C*_away_,*M*_away_) responses (effects of attention on centre and surround/mask responses predicted from attend away centre/surround responses) for each of the 12 different centre orientations.

We performed χ^2^ error minimization for the 60 different responses (five predictions as outlined above for 12 different centre stimulus orientations each). The models were able to accommodate the hypothesis that the focus of attention was narrow for all stimulus conditions (i.e. affecting only the drive centre stimuli yield), or that the focus of attention was wide for all stimulus conditions (i.e. affecting the drive that centre stimuli yield and the drive that surround stimuli yield). However, it might be assumed that the animals would alter their focus of attention depending on the stimulus condition, whereby they use a wide focus of attention when centre only stimuli were presented, while they use a narrow focus of attention when centre and surround stimuli were present. This hypothesis was also accommodated by one set of models, whereby it required us to alter the value for c_2_, when no surround stimulus was present as the narrow focus of attention and the variable focus of attention models would otherwise be identical. To test for the validity of the variable attention focus model it was set to 0.01 when no surround was present. Overall, this approach yielded 18 different models [three different multiplicative models, three different additive models, for three conditions each (wide, narrow and variable attention field)].

To determine which model best explained our data we calculated Akaike's information criterion (AIC). Under the assumption of independent normally distributed errors, AIC corresponds to (Burnham & Anderson, 2004):


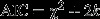
(10)

where χ^2^ is the summed squared error across the fitted data for each neuron and model, and *k* corresponds to the number of free parameters in the model, i.e. 1 and 2, respectively. This yielded AIC1 to AIC18 for the 18 models. Final model comparison was based on Akaike weights (*w*_i_):


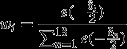
(11)

Here, δ_i_ corresponded to AIC_i_ – AIC_min_, where AIC_min_ was the smallest AIC obtained for the 18 model fits. The larger the *w*_i_, the more evidence existed in favour of model *i*.

The Bayesian information criterion (BIC) is often used instead of AIC. BIC applies a larger penalty on free parameters in the model and is calculated according to


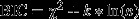
(12)where χ^2^ is the summed squared error across the fitted data for each neuron and model, *k* corresponds to the number of free parameters in the model, i.e. 1 and 2, respectively, and n corresponds to the number of data points (i.e. 60, as we aimed to predict five different conditions with 12 different responses each). The BIC weights are calculated as in Eqn (12). The larger the BIC weight, the more evidence existed in favour of a given model.

### Eye position analysis/control

We calculated the mean *x* and *y* eye position for each stimulus (centre only, centre surround, surround only) and attention condition [attend away/attend RF for the period of interest (100–500 ms after stimulus onset; we started at 100 ms, to ensure that differential eye movements shortly before analysis start did not affect our data)]. For each neuron recorded (i.e. for each recording session) we calculated a grand mean *x*-position and a grand mean *y*-position (across all stimulus conditions; correct trials only) and subtracted these grand mean values from the mean single trial *x*- and *y*-position. This procedure eliminates potential differences in eye-position calibration between recording sessions, and thus allows comparison across sessions. We performed separate two-factor anovas on the *x*- and *y*-position eye-movement data to determine whether mean eye-position in *x* or in *y* differed between the attention and stimulus condition. We found no significant difference in mean *x*-position between different attention conditions (effect of attention: *P* = 0.623; effect of surround: *P* = 0.974; interaction: *P* = 0.893). Similar results were obtained for the *y*-position eye data (effect of attention: *P* = 0.348; effect of surround: *P* = 0.865; interaction: *P* = 0.993). This suggests that differences in mean eye position did not contaminate our analysis.

## Results

We recorded 105 neurons in two monkeys (62 in monkey HU, 43 in monkey HO). Post-mortem histological analysis confirmed that recordings from intended V1 craniotomies were indeed in V1 in both animals.

Neuronal tuning width, tuning amplitude and tuning baseline were determined by fitting the neuronal responses with a wrapped Gaussian. The investigation of fitted data was restricted to neurons that were reasonably well described by the wrapped Gaussian function as assessed by our exclusion criterion (variance accounted for, orientation index and maximum response, see Methods). After exclusion of poorly fitted responses we were able to analyse 70 neurons (38 from monkey HU, 32 from monkey HO) in this part of the study, while we used all 105 cells to investigate whether normalization models account for attention effects in our study.

**Figure 2:**
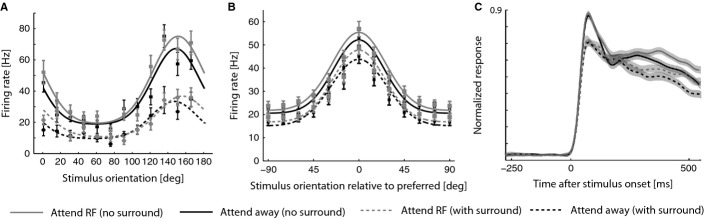
(A) Effects of attention and surround stimulation on orientation tuning in an example cell. Filled squares and circles show the mean activity (*y*-axis) recorded given the stimulus orientation (*x*-axis), and solid continuous lines show the wrapped Gaussians fitted to these data points. Attention (grey lines) increased the neuronal activity when stimuli were close to the preferred orientation. Surround stimuli reduced the neuronal activity at all orientations (dashed lines). Error bars show SEM. (B) Effects of attention and surround stimulation on orientation tuning on the population of cells. Solid fitted lines show the effects when no surround stimuli were presented, and dashed lines show the effects when a surround stimulus was presented. (C) Normalized population PSTHs when no surround stimulus was presented (solid lines) and when a surround stimulus was presented (dashed lines). Shaded areas show SEM. Time zero corresponds to stimulus onset. Grey lines show the neuronal activity when stimuli were attended to, and black lines when attention was directed into the opposite hemifield.

An example cell that was well fitted with the wrapped Gaussian in the four conditions (attend RF/attend away; without surround/with surround) is shown in Fig.2A. The cell preferred an orientation of ∽ 150°, it showed stronger responses in the attend RF condition when the preferred stimulus was presented, and its responses were strongly suppressed by the surround stimulus.

Surround stimuli reduced the tuning amplitude for the example cell shown in Fig.2A. The effects of surround stimuli on baseline and tuning width were less clear from the single cell example shown in Fig.2A. To establish the effects of surround stimuli at the population level, we fitted each cell's response as shown in Fig.2A, whereby the preferred orientation in the attend away – no surround condition was set to be at 0°, and arranged all other orientation related responses accordingly. The population-averaged responses are shown in Fig.2B. To provide an impression of the effects of surround stimulus presentation and attention as a function of time after response onset we show the normalized PSTH for preferred orientation responses in Fig.2C. Figure2B and C show that surround stimuli reduced the overall activity. Attention, conversely, increased the activity. From Fig.2C it is apparent that attention effects are more prominent during the later response period, while surround effects are prominent during early and late response periods. Given our interest in the interaction between attention and surround effects, we focus on the late response period for the remainder of the paper.

**Figure 3:**
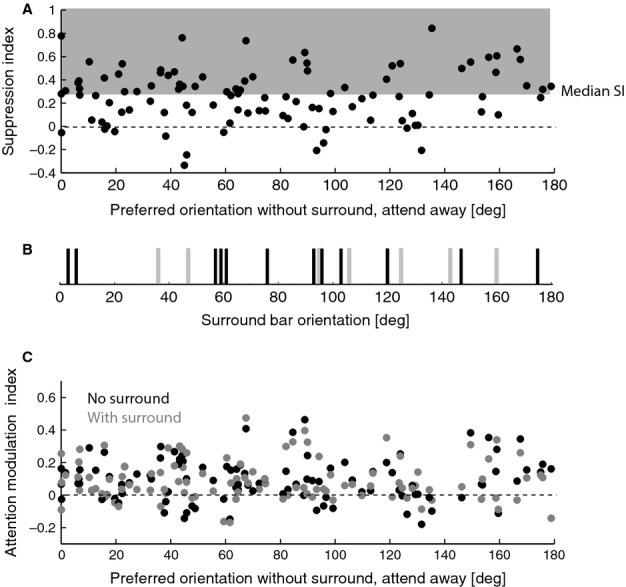
(A) Suppression index (SI) plotted against the preferred orientation of a given cell. Preferred orientations in the cell sample were relatively uniformly distributed (spread of points along the *x*-axis in the upper graph). SIs were mostly positive, i.e. in most cells surround stimuli reduced the neuronal activity. (B) Orientation of the bars in the surround. Grey tick marks show the orientation of bars on the inner ring, and black tick marks show the orientation of bars on the outer ring of the surround stimulus. (C) Attentional modulation indices plotted against the preferred orientation of a given cell. Attentional modulation indices were mostly positive, i.e. in most cells attention increased neuronal activity. Neither suppression nor attentional modulation indices showed an obvious relationship to preferred orientation of the neurons, or to the distribution of orientations in the surround stimulus.

The surround stimulus used was identical for all cells. The specific distribution of orientations present in the surround might therefore yield different effects on neurons, depending on their preferred orientation. This argument is based on the established differential effects of iso- and cross-orientation inhibition (Sengpiel *et al*., 1998; Jones *et al*., 2001, 2002; Freeman *et al*., 2002). Therefore we calculated the suppression index (SI) for each neuron and plotted this against the neuron's preferred orientation (Fig.3A). Neurons with stronger SI (larger than the median of the population) showed no obvious clustering around specific orientations, and there was also no obvious relationship between strength of SI, preferred orientation of a neuron and clustering of surround orientations (Fig.3B).

The relationship between the features of the distracter and the neuronal tuning for the attended stimulus (feature-based attention) influences the effects of attention/normalization for area MT neurons (Khayat *et al*., 2010). While Fig.3A addresses the question of whether there was a relationship between the tuning of the neurons, the stimuli in the surround and the strength of normalization in our study, it does not address whether there is a relationship with the strength of attentional modulation. This is addressed in Fig.3C, where attentional modulation indices are plotted in relation to the preferred orientation of neurons, along with the distribution of surround orientations (shown above in Fig.3B). Neurons with stronger attentional modulation showed no obvious clustering around specific orientations, and there was also no obvious relationship between strength of MI, preferred orientation of a neuron and clustering of surround orientations. This is not really surprising, as our task was a spatial attention one, not a feature-based attention task, and only the latter shows systematic influences of different stimulus features on attentional modulation (Treue & Trujillo, 1999; McAdams & Maunsell, 2000).

To determine whether any of the surround- and attention-induced changes seen in Fig.2 were significant we used the three different parameter estimates from the wrapped Gaussian fitting for each cell under the four different conditions (attend away – attend RF; no surround – with surround) and subjected them to a two-factor repeated-measure anova with *attention* and *surround* as the two factors. The results are given in Table1. In short, neither the surround nor attention had a significant effect on the tuning widths, attention significantly increased the tuning amplitude and baseline (*P *<* *0.05, Table1), while the surround significantly reduced the tuning baseline (*P *<* *0.05, Table1). The effects, as described above, were significant when assessed for each of the two monkeys individually (*P *<* *0.05, two-factor repeated-measure anova).

**Table 1:** Effects of surround stimuli and of attention on amplitude, baseline and half width-half heights (HWHH) of the tuning functions *n* = 70

	Attend away (median [25% 75%])	Attend RF (median [25% 75%])	*P*(surr.)	*P*(att.)	*P*(surr^*^att.)
HWHH					
No surround	24.21 [15.79 34.80]	23.98 [18.02 35.85]	0.880	0.732	0.404
Surround	23.58 [16.16 37.85]	24.11 [15.52 32.89]			
Amplitude					
No surround	21.27 [13.32 39.69]	26.70 [12.32 41.18]	0.631	0.018	0.654
Surround	22.26 [11.70 33.53]	23.00 [15.33 37.90]			
Baseline					
No surround	17.90 [11.62 29.54]	19.22 [11.32 31.29]	< 0.001	0.002	0.772
Surround	12.27 [7.80 22.18]	14.24 [8.16 26.26]			

The values in the left two columns show the median [25/75 percentiles] of the respective value distributions. The three columns to the right show *P*-values (two-factor repeated-measures anova) relating to the effects of surround stimuli (surr.), of attention (att.) and the possible interaction between the two factors (surr^*^att.).

It could be argued that the effects described above were a consequence of ‘direct’ surround intrusion, whereby the RFs of the cells extended into the area that was covered by the surround stimulus. To account for that possibility we restricted our analysis to cells that were not directly affected by the ‘surround only’ stimulus condition. We also performed the surround control measures as described in the Methods (i.e. surround only activity subtracted). This control reduced our cell sample to 36 cells, but it did not change the basic results. For this reduced sample size, the surround stimulus reduced the tuning baseline (*P *<* *0.001), and there was a trend towards a reduction of the tuning amplitude (*P* = 0.083). Attention significantly increased the tuning amplitude (*P *<* *0.05), and it showed a trend towards increasing the tuning baseline (*P* = 0.095). The changes in significance are probably accounted for by the reduced sample size. Thus, the effects described are not a consequence of direct surround intrusion.

**Figure 4:**
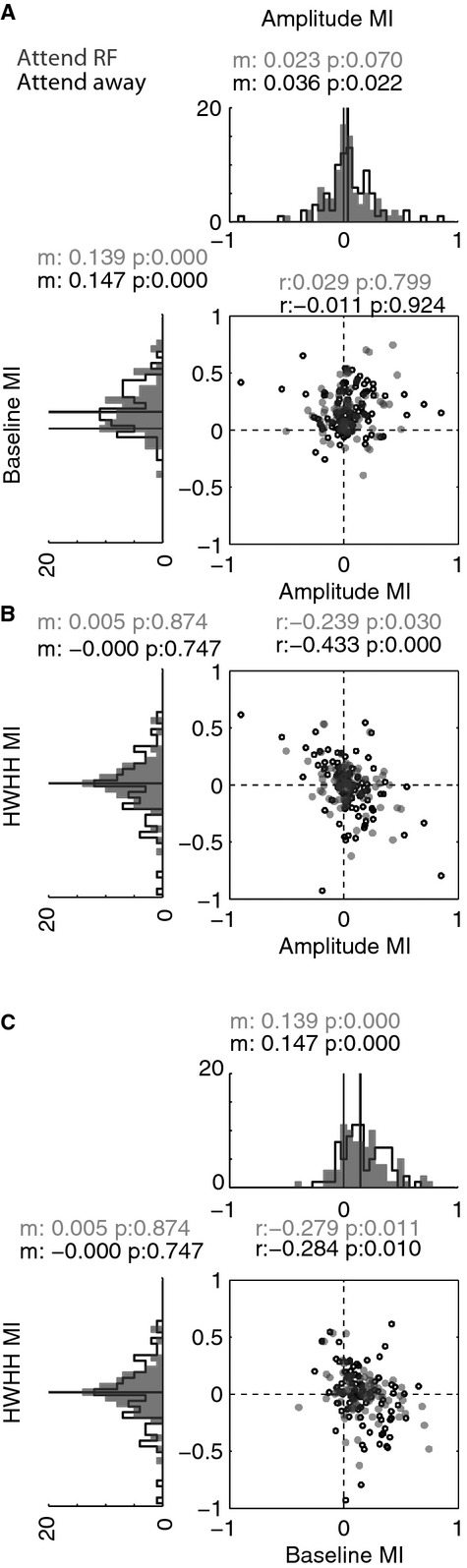
Effects of surround stimuli on tuning amplitude, baseline and bandwidth of the wrapped Gaussian fitted to the responses of each cell. Effects are shown separately for the attend away (black) and the attend RF (grey) condition. Centre plots (and associated text: *r* = correlation coefficient, *P* = significance of correlation) show whether modulation indices (MIs) were correlated between amplitude, baseline and tuning width. Histograms above and to the side of centre plots show distributions of MIs. Associated text indicates the median of these distributions and whether these were significantly different from 0 (associated *P*-value).

The above analysis directly used the values of the fitted wrapped Gaussian parameters as input variables. A corresponding picture emerges when analysing modulation indices (MIs of fitted parameters) instead. The MI distributions are plotted in Fig.4. Surround stimuli had a limited effect on tuning amplitude (Fig.4A, top histogram). The distributions for the attend RF conditions were not different from zero (*P *>* *0.05, Wilcoxon sign rank test), but the MI distribution for the attend away condition was significantly larger than zero. Surround stimuli significantly reduced tuning baseline for both attention conditions (Fig.4A, rotated histograms, distributions were significantly larger than zero, *P *<* *0.05, Wilcoxon sign rank test). Surround stimuli did not have systematic effects on the tuning width (Fig.4B, rotated histograms), i.e. the median tuning width MI was close to zero and the distributions were not significantly different from zero (*P *>* *0.05, Wilcoxon sign rank test). The effects described were also found when the ‘surround only’ stimulus-induced activity was subtracted from the surround plus centre stimulus conditions, and when neurons that exhibited significant activity changes upon ‘surround only’ stimulus presentation were excluded from the sample (see Methods). Thus, the main effect of surround stimulus presentation was an overall reduction in stimulus-driven activity, irrespective of the stimulus orientation, i.e. an offset effect, which was reflected in the overall reduced tuning baseline.

Previous studies have reported that surround-dependent suppression of tuning amplitude was positively correlated with surround effects on tuning bandwidth (Chen *et al*., 2005; Okamoto *et al*., 2009). In those studies surround stimuli sharpened tuning curves and cells that were most strongly suppressed by surround stimuli showed the most profound sharpening. To determine whether such a relationship could also be found in V1 of the awake macaque (and/or whether other correlations existed), we calculated the correlation (Methods) between surround-dependent amplitude MI and the surround-dependent tuning width MI (Fig.4B), between surround-dependent amplitude and surround-dependent baseline MI (Fig.4A), and between surround-dependent baseline MI and tuning width MI (Fig.4C). The surround-dependent amplitude MI was not correlated with the baseline MI, but the amplitude and tuning width were negatively correlated in the attend away and the attend RF condition (*P *<* *0.05, Spearman correlation). Baseline MI and tuning width MI were also significantly negatively correlated (*P *<* *0.05, Spearman correlation). The negative correlations were present in each monkey individually (*P *<* *0.05, Spearman partial correlation). The above described significant negative correlations were equally found when the ‘surround only’ stimulus-induced activity was subtracted from the surround plus centre stimulus conditions and neurons with significant activity changes upon ‘surround only’ stimulus presentation were excluded from the sample. Thus, if surround stimuli induced a reduction in tuning amplitude (or baseline) then they usually also caused a widening of the tuning curve. Conversely, if surround stimuli induced an increase in tuning amplitude (or baseline), then they usually also caused a sharpening of the tuning curve. Note that an increase in tuning amplitude does not necessarily imply an increase in neuronal activity with surround presentation, as the tuning amplitude can increase if the presence of a surround reduces the tuning baseline more profoundly than it reduces the tuning amplitude.

**Figure 5:**
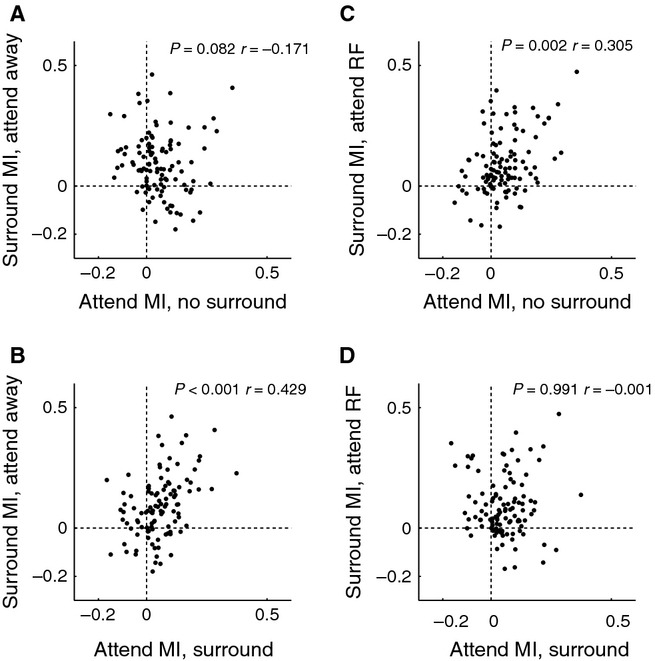
Correlation of attentional and surround modulation index (based on raw firing rates averaged for the preferred stimulus orientation). Four different comparisons were possible which are shown in A–D, respectively. Specifically, we obtained an attentional MI when no surround stimulus was present and when a surround stimulus was present. Moreover we obtained a surround MI when attention was directed away from the RF and one when it was directed towards the RF. Text insets report the correlation coefficient (*r*) and the significance of the correlation (*P*).

So far we have investigated how the different tuning parameters are affected by surround stimulation and attention, and whether changes in a given parameter were correlated with changes in a different parameter. We now turn to the question of whether the effects of attention and of surround stimulation on the neuronal activity were correlated. This might suggest that attention and surround suppression use a similar mechanism, as proposed by normalization models of attention. A recent study reported that strength of normalization was correlated with the strength of attentional modulation, when attention was shifted between preferred and anti-preferred stimuli that were placed within the RF of MT neurons (Ni *et al*., 2012), in fact a condition more similar to masking than surround suppression. Here we explore whether similar correlations can be found for V1 neurons. RFs in area V1 are usually too small to allow for placement of separate stimuli inside the RF and for differential allocation of attention to these two stimuli. For this reason, our attentional modulation index is based upon attention away from the RF and attention inside the RF conditions and the normalization index is based on data acquired when the RF centre was stimulated and when the RF centre and the RF surround were both stimulated. For this analysis we used the entire neuronal sample (*n* = 105). We used two approaches to calculate the MIs. For the first approach we only used the preferred orientation responses, while for the second we used the average response to all 12 orientations. From these responses attention MIs and normalization MIs were obtained. We found that the correlation for one of our comparisons depended on the approach taken to calculate MIs. Using MIs calculated from preferred orientation responses only, we found a trend for a negative correlation between attention MI – no surround and surround – attend away MIs (Fig.5A, *P* = 0.082 *r* = −0.171). This is contrary to predictions from attentional normalization models. However, using the same MI types calculated from responses to all bar orientation yielded a trend for a positive correlation between the surround MI – attend away condition and the attention MI – no surround (*r* = 0.179, *P* = 0.068, Spearman rank correlation, data not shown), results somewhat reminiscent of those described previously for MT neurons (Ni *et al*., 2012), even though the effect in these data was much smaller (and only approaching significance). Other comparisons were more consistent across responses used to calculate MIs. We found a significant positive correlation between the surround MI in the attend away condition and the attention MI when a surround was present (*P *<* *0.001, *r* = 0.429, Spearman rank correlation, Fig.5B preferred orientation responses; *P *<* *0.001, *r* = 0.309, responses to all bar orientations, data not shown). Moreover, surround MIs (measured when attention was directed to the RF) were positively correlated with attention MIs measured in the absence of surround stimuli (*P* = 0.002, *r* = 0.305, Spearman rank correlation, Fig.5C preferred orientation responses; *r* = 0.334, *P *<* *0.001, responses to all bar orientations, data not shown). No correlation between surround MIs measured in the attend RF condition and attention MIs measured when surround stimuli were present was found (*P* = 0.991, *r* = −0.001, preferred orientation response MIs, Fig.5D; *P* = 0.819, *r* = −0.023, MIs from responses to all bar orientations, data not shown, Spearman rank correlation). To summarize, correlations between surround suppression indices and attentional modulation are at best weak to moderate. Some of our data support a normalization model of attention, but the latter would have predicted positive correlations between attentional MIs and surround MIs for all comparisons, which we did not find, and notably not for a critical comparison as shown in Fig.5A.

To further explore the relationship of our data to normalization models of attention further, we fitted our data to attention–surround models that were based on current discussions about the effects of attention on neuronal activity (but note that these models are by no means exhaustive). We decided to use two main types of models (additive vs. multiplicative) based on previous reports about the effects of attention on neuronal activity for single stimuli inside the RF in striate and extrastriate cortex (McAdams & Maunsell, 1999; Thiele *et al*., 2009). Given the results shown in Fig.5, we adopted each of the models to either allow or not allow attention to contribute to normalization. The model that assumes attention to contribute to normalization was further subdivided into two models based on the results reported by Ni *et al*. (2012), whereby attention and surround stimulation either used exactly the same mechanisms (single parameter model) or they were allowed to use separated mechanisms (two-parameter models). This resulted in six models which were based on current discussions about the effects of attention on neuronal activity, without intending to imply that there are no other models that are used to explain attentional effects. Each of these six models could then either assume that the attentional focus was narrow for all stimulus conditions (whereby the attentional scaling term β would only affect centre stimulus responses), or that the attentional focus was wide for all stimulus conditions (whereby the attentional scaling term β would also affect surround/mask responses), or finally that the attentional focus was wide when centre only stimuli were presented, while it was narrow when surround stimuli were also presented. This resulted in a total of 18 models that were initially fitted to our data. For this analysis we included all 105 cells, as these models do not make assumptions about the responses of the cells to different stimulus orientations, and thus responses did not need to be accounted for by wrapped Gaussians. We also did not exclude neurons based on analysis of surround intrusion, as previous studies intentionally placed the second stimulus inside the RF, which would be complete surround intrusion.

The models assuming narrow fields of attention consistently resulted in better fits [and thus larger Akaike (or BIC) weights] than models assuming either variable or wide fields of attention. Specifically, 60/105 neurons were best fit with a narrow focus of attention model, 32/105 were best fit with a variable focus of attention model and 13/105 neurons were best fit with a wide focus of attention model. Using models for all three focal attention sizes, we found that the majority of cells were best described by an additive model which assumes that attention does not contribute to normalization (59/105), with a further ten cells which were best described by a multiplicative model which assumes that attention does not contribute to normalization mechanisms, Thus, 69/105 cells were best described by models where attention does not contribute to normalization. While it is possible that monkeys used different attentional strategies in different sessions (and thus different neurons would need to be fit with different basic models), we assume it is more parsimonious that monkeys used the same approach throughout, and therefore applied a single fitting approach (narrow focus of attention which yielded the best fit for the majority of cells) for model comparison. We thus excluded wide and variable attentional focus models from further analysis (i.e. we re-ran our analysis based on narrow attention field assumptions only). Importantly, this reduction in model numbers has no effect on our main conclusions that we draw from the data in the next few sections (compare, for example, the number of cell best fitted with models where attention does not contribute to normalization with those presented in Table2). Using the narrow field attentional focus model exclusively meant that the attentional scaling term β only affected centre stimulus responses (i.e. the definition of narrow attentional focus). Table2 gives an overview of the different narrow field models and the assumptions they make regarding the size of the attentional field.

**Table 2:** Overview of the different small focus of attention models tested for model selection

Model number	Model class	Attention normalization model	No. of free parameters	Size of attentional field	Mean weight	No. of cells with max. Akaike weight [BIC weights]
1	Multiplicative	Yes	1 (attention effect fixed = 1.37)	Small	0.035 ± 0.098	5 [10]
2	Multiplicative	Yes	2	Small	0.119 ± 0.264	12 [11]
3	Multiplicative	No	2	Small	0.157 ± 0.357	10 [10]
4	Additive	Yes	1 (attention effect fixed = 7.39)	Small	0.002 ± 0.021	0 [1]
5	Additive	Yes	2	Small	0.184 ± 0.267	19 [19]
6	Additive	No	2	Small	0.501 ± 0.440	59 [54]

Models 1–3 assume that attention affects responses in a multiplicative manner, and models 4–6 assume that the effects are additive. Models 1, 2, 4 and 5 assume that attention also contributes to normalization. Models 1 and 4 had a single free fitting parameter, which allowed the strength of normalization to vary, while the effect of attention was fixed for all neurons as given in the table. Column 6 lists the mean [±SD] Akaike weights for the specific model, while the seventh column lists the number of neurons that gave the largest weight given the specific model. Square brackets indicate the respective numbers when BIC weights are used, rather than Akaike's weights.

**Figure 6:**
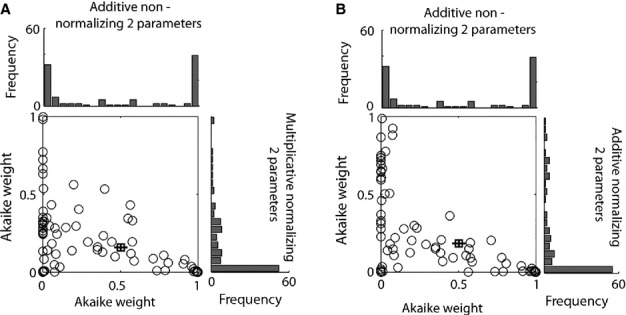
Pairwise distribution of Akaike weights for three different models. (A) Distribution of Akaike weights for the additive non-normalizing two-parameter model (*x*-axis) vs. the additive normalizing two-parameter model. The average weight (±SEM) for both models is indicated by the location of the square and error bars. (B) Distribution of Akaike weights for the additive non-normalizing two-parameter model (*x*-axis) vs. the multiplicative non- normalizing two-parameter model. The average weight (±SEM) for both models is indicated by the location of the square and error bars. Histograms along the axes show the frequency distributions.

To decide which model best captured our data, we re-determined Akaike (and Bayesian) weights based on these six models only (see Methods). We counted how often a given model had the largest evidence (when compared with the other models). The number of cells best described by a particular model (using AIC and BIC weights) is listed in Table2. Given that AIC and BIC yielded almost identical results we will focus on AIC results. Most neurons were best described by a model where attention acted in an additive and non-normalizing manner, followed by an additive model where attention does contribute to normalization. The pairwise distribution of weights comparing the two best models (additive non-normalizing vs. additive normalizing) is shown in Fig.6A, and the pairwise distribution of weights comparing the best and the third best model (additive non-normalizing vs. multiplicative normalizing) is shown in Fig.6B. Even the best model yields Akaike weights that are close to zero for a fairly large number of cells (see frequency histograms along the *x*-axes in Fig.6). However, this does not necessarily mean that the model poorly fitted the data, but simply that another model yielded a substantially better fit. In summary, most cells were better described by additive models (74.3%), when compared with multiplicative models. Moreover, 65.7% of cells were best described by models where attention does not contribute to normalization mechanisms.

To test whether the above reported data would also hold for conditions where fitting was entirely based on attend RF vs. attend away conditions (i.e. ignoring the fits where the effects of surround/mask stimuli were estimated under constant attention conditions), we refitted our data for just three conditions, namely attend RF rates predicted from attend away – no surround conditions, and attend RF rates predicted from attend away – with surround/mask conditions (two versions as outlined in Methods). These fits yielded similar results with respect to the effect of attention on normalization, Under these more constrained fitting conditions, 60/105 cells were best supported by the two-parameter attention additive non-normalization model, while 23/105 were best described by a multiplicative non-normalization model (i.e. 79.5% of cells were best described by a model where attention does not contribute to normalization). Fifteen cells were best described by a two-parameter additive normalization model (i.e. 71.4% of cells were best described by an additive model under conditions when the effect of surround stimulations is not part of the fitting routine). Finally, we restricted our fitting to the conditions where we predict attend RF – no surround responses from attend away – no surround responses (i.e. excluding any surround modulation). Under these conditions, 60/105 cells were best supported by a two-parameter attention additive non-normalization model, 19/105 cells were best supported by the two-parameter multiplicative non-normalization model and 17/105 cells were best described by a two-parameter additive normalization model (i.e. 75.2% of cells were best described by a model where attention does not contribute to normalization). These controls demonstrate that the results do not simply arise from specific fitting selections.

**Figure 7:**
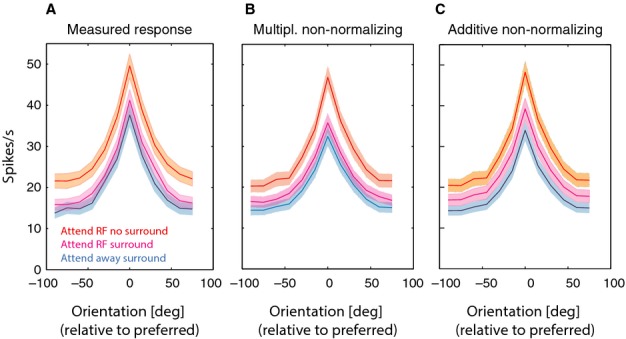
Comparison of measured against predicted responses. (A) Population average of measured responses. Red line shows the mean population response in the attend RF – no surround condition (shaded area shows SEM). The magenta line shows the attend RF – with surround responses, and the blue line shows the attend away – with surround responses. (B) Predicted responses from the multiplicative non-normalizing model (colour coding as in A). (C) Predicted responses from the additive non-normalizing model (colour coding as in A).

While the above data show the relative quality of fits when compared among the different models, they do not provide insight into the absolute quality of the fits. It might be that none of the models describe the data well, and therefore account for little of the variance. Figure7 shows that this was not the case. It displays the measured population responses (Fig.7A) and the fitted population response for the two non-normalizing models [multiplicative (Fig.7B) and additive non-normalizing models (Fig.7C)]. Across the population of 105 cells the two models fitted the data well. The multiplicative non-normalization model accounted for 94.1% of the variance (across all five fitting conditions) while the additive non-normalization model accounted for 96.4% of the variance (across all five fitting conditions). It is noteworthy, however, that our models, despite accounting for most of the variance, underestimate the peak amplitude response for some of the conditions by about 5–10% (see Fig.7), which was less pronounced for the additive non-normalizing model (Fig.7B). This raises the question of whether normalization models of attention might account for the data, if fits were (i) constrained to preferred orientation responses only or (ii) constrained to responses excluding preferred orientation responses. Preferred orientation responses here are simply defined as the responses that yielded the largest activity in the attend away no surround condition, as defined above. Given that the analysis included all neurons, not just those that could well be described as orientation tuned under all stimulus conditions (*n* = 70, see above), it may include neurons where a genuine preferred orientation cannot be delineated. But given the results shown in Fig.7A, even including the remaining 35 neurons yields clear orientation-tuned population responses. And because the exclusion approaches described above simply serve as a control we believe this to be a viable approach. Fitting our data using either approach yielded similar results to those described above. In 54/105 neurons support was obtained for the additive model where attention does not contribute to normalization, and in 13/105 neurons support was obtained for the multiplicative model where attention does not contribute to normalization. Fitting our data using all responses except preferred orientation responses supported the additive model where attention does not contribute to normalization in 66/105 neurons, and the multiplicative model where attention does not contribute to normalization in 6/105 neurons. Thus, the under-fitting of preferred orientation responses is not the cause of the general results reported here.

**Figure 8:**
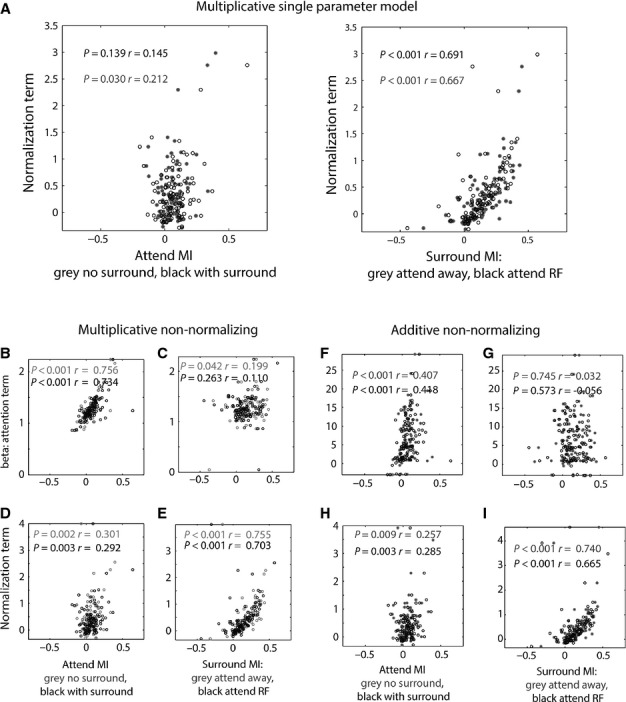
Correlation of fitting parameters with modulation indices. (A) Correlation between the attentional and normalization fitting parameter from the multiplicative model (single parameter model) and measured attention and surround modulation indices. (B–E) Correlation between the attentional and normalization fitting parameters from the multiplicative non-normalizing two-parameter model with the measured attention and surround modulation indices. (F–I) Correlation between the fitting parameters from the best fitting additive attention non-normalizing two-parameter model and measured attention and surround modulation indices. The normalization term captures surround effects, while the parameter β captures attention effects. Text insets give correlation coefficients (*r*, Spearman rank correlation) and significance (*P*). Grey and black symbols/text delineate different MI conditions as labelled along the *x*- and *y*-axes.

We next tested whether the attention modulation term (β) and the surround modulation term (α) for the additive non-normalization model were correlated. We found no correlation between these parameters, irrespective of whether the parameters of all cells were used (fitting the additive non-normalizing model to the data, *r* = –0.020, *P* = 0.843), whether we only used the cells that were best fitted by the additive non-normalizing model (*n* = 59, *r* = −0.033, *P* = 0.805) or whether the parameters were used obtained from whichever model best fitted a given cell (*r* = −0.139, *P* = 0.155). The absence of a correlation between β and α has previously been used as an argument against such a two- (independent) parameter model (Ni *et al*., 2012). The rationale for this argument is based on the result that attentional modulation indices and surround modulation indices were correlated, and so a correlation between fitted parameters would in principle be expected. As we equally did not find a correlation between the attention modulation term (β) and the surround modulation term (α) in the best model, we followed the rationale of Ni *et al*. (2012) and employed single parameter models (additive normalizing, multiplicative normalizing) to fit our data. These models usually resulted in worse fits and thus reduced Akaike weights (Table2); in fact, they yielded the worst fits when compared with any of the two-parameter models applied. This is despite the fact that the single parameter additive normalizing model resulted in significant correlations between the free normalization/attentional scaling parameter and the attentional and normalization modulation indices as measured (Fig.8A).

Figure8B–E show the correlation of α and β derived from the multiplicative non-normalization model with the attentional MIs and with the surround MIs, respectively. As expected the attention term β was significantly correlated with attentional MIs, and the surround normalization term was significantly correlated with the surround MIs. The attention term β was weakly correlated with the surround modulation MIs for the attend RF condition, but not in the attend away condition. The surround normalization term α was correlated with the attention MIs. A similar pattern was found for the additive non-normalization model as shown in Fig.8F–I, except for the relationship between the attention term β and the surround MI, where no correlations were found.

## Discussion

Here we report the effects of attention- and of surround/mask-induced normalization on V1 orientation tuning functions, and explore to what extent various attentional models account for our data. We found that attention increased the amplitude and the baseline of orientation tuning functions in macaque V1 neurons, while having little effect on the average tuning width. Surround/mask stimuli significantly reduced the baseline, but did not affect the tuning amplitude or average tuning width. Despite the opposite effects of attention and surround stimulation, their effect at the single cell level was still correlated, i.e. attentional MIs and surround MIs often showed a positive correlation. Normalization models of attention predict such a correlation, as attention and surround stimuli will affect the same neuronal circuit. However, model selection based on AIC revealed that the majority of cells (60–74% depending on the exact fitting approach) were best described by multiplicative and additive attention models which did not assume attention to contribute to normalization.

### Effects on tuning amplitude

Attention increases the amplitude of neuronal tuning functions in striate (Spitzer *et al*., 1988; McAdams & Maunsell, 1999; Roberts *et al*., 2007; Thiele *et al*., 2009) and extrastriate cortex (Moran & Desimone, 1985; Spitzer *et al*., 1988; Treue & Maunsell, 1996, 1999; Martinez Trujillo & Treue, 1999; McAdams & Maunsell, 1999, 2000; Martinez-Trujillo & Treue, 2004; Williford & Maunsell, 2006). Thus, our result of increased tuning amplitude when attention was deployed to the neuron's RF was predicted. Surround stimuli generally reduce tuning amplitude in striate and extrastriate areas (Li *et al*., 2000; Walker *et al*., 2000; Angelucci *et al*., 2002; Cavanaugh *et al*., 2002b; Bair *et al*., 2003; Ozeki *et al*., 2004; Chen *et al*., 2005; Xing *et al*., 2005; Okamoto *et al*., 2009; Bartolo *et al*., 2011), and our results are also in line with those data. While attention and surround stimuli have opposite effects on the amplitude of the tuning function at the population level, normalization models of attention (Lee & Maunsell, 2009; Reynolds & Heeger, 2009) predict that surround modulation indices and attentional modulation indices are correlated across the population and that the effects of attention and surround stimuli can be adequately captured by normalization models. By and large we found that cells most strongly suppressed by surround/mask stimuli yielded the strongest increase in activity when spatial attention was directed to the cell's RF.

### Effects on tuning width

Previous studies have reported cells that were most strongly suppressed by surround stimuli showed the most profound sharpening of tuning curves (Chen *et al*., 2005; Okamoto *et al*., 2009). We found the opposite in our study. A sharpening of tuning functions could in principle be a ‘tip of the iceberg’ effect. If surround stimuli reduce firing rates for non-preferred orientation stimuli to zero, a fit to those data may yield ‘apparently’ more narrow tuning curves, as cells cannot exhibit negative firing rates. But whether this contributes to the discrepancy between our and previous data, or whether it is a result of anaesthesia or species differences is currently undetermined.

### Effects on tuning baseline (asymptote)

Surround stimuli reduced the tuning baseline, which is in line with previous studies (Li *et al*., 2000; Walker *et al*., 2000; Angelucci *et al*., 2002; Cavanaugh *et al*., 2002b; Bair *et al*., 2003; Ozeki *et al*., 2004; Chen *et al*., 2005; Xing *et al*., 2005; Okamoto *et al*., 2009; Bartolo *et al*., 2011), and this was independent of whether the surround stimuli encroached upon the ‘classical RF’. Attention increased the tuning baseline in V1, which differs from previous reports in area V1 (McAdams & Maunsell, 1999) and area MT (Treue & Trujillo, 1999), where the asymptote of the tuning function was not affected. In our data the effect was only significant when all neurons were analysed. When neurons with surround intrusion were removed from the sample, the effect on tuning baseline was no longer significant, which we assign to the reduced sample size. This leaves the question of why we found changes in tuning baseline with attention, while the above studies have not. It could be related to differences in the visual stimuli used, as McAdams & Maunsell (1999) used Gabor stimuli, which usually will have stimulated the RF centre in full, but also encroached into the surround, but it is unclear whether that is sufficient to explain the differences.

### Effects of attention: additive or multiplicative?

A previous study argued that the effects of attention in area V1 are best described by an additive model (Thiele *et al*., 2009), which is in contrast to other cortical areas, where multiplicative effects (Williford & Maunsell, 2006) or contrast gain changes (Reynolds *et al*., 2000) have been reported. Our modelling (based on the full set of fitting procedures, see Methods) supports the notion that effects of attention in V1 are best described by additive models, as the largest number of neurons was best described by these models. However, a sizeable number of neurons were best explained by multiplicative models, which demonstrates that both effects can be present intermixed within an area.

### Normalization models of attention

Normalization models have successfully explained visual responses under a variety of stimulus conditions (Heeger, 1992; Carandini *et al*., 1997; Tolhurst & Heeger, 1997; Britten & Heuer, 1999; Anderson *et al*., 2000; Sceniak *et al*., 2001; Thiele *et al*., 2004; Rust *et al*., 2006). These models assume that tuned local excitatory inputs are normalized by the sum of inputs, whereby the ‘normalizing’ inputs can be tuned themselves (Ni *et al*., 2012). Normalization models have more recently been used to successfully explain conflicting results regarding the influence of attention on neuronal tuning functions (Lee & Maunsell, 2009; Reynolds & Heeger, 2009), and they have gained experimental support from human psychophysics and functional magnetic resonance imaging (Herrmann *et al*., 2010), and single cell data (Lee & Maunsell, 2010; Ni *et al*., 2012). A key prediction of the normalization model of attention is that presentation of surround stimuli and deployment of attention to the RF should have correlated effects. A recent paper provided evidence in favour of this prediction, employing normalization models to explain the effects of attending to one of two stimuli that are presented inside the RFs of MT neurons (Ni *et al*., 2012). The authors reported a correlation between the strength of normalization modulation and attentional modulation (their Fig. 3). We also found a correlation between the strength of normalization modulation and attentional modulation for some of our comparisons (see Fig.5), which provides partial support for the normalization model of attention. However, a key comparison showed a trend towards a negative correlation (using preferred orientation responses) for the condition which could be viewed as showing ‘pure’ effects of surround influences and pure effects of attention, i.e. when the surround MI (attend away) was compared with the attend MI (no surround). Using the responses from all 12 bar orientations to calculate MIs for this comparison, by contrast, yielded a weak positive correlation. This negative (or weak positive) correlation for the most ‘pure’ effects questions the link between attention and normalization for our sample, but it is noteworthy that we did not investigate tuned normalization (as Ni *et al*. did), but rather a form of un-tuned normalization. Ni *et al*. (2012) furthermore found a correlation between parameters from their fitted normalization models and attention, as well as between parameters from their fitted normalization models and normalization modulation indices. We also found that the derived parameter from a multiplicative attention normalizing model was significantly correlated with attentional and with surround modulation indices. However, model validation based on AIC (and BIC) weights (Akaike, 1992; Burnham & Anderson, 2004) suggests that this particular model yielded a relatively poor description of the data, when compared with other models. In most cases an additive attention model, where attention did *not* contribute to normalization mechanisms, was the best model (in relative terms) for our data. We emphasize the fact of ‘relatively’ best model, as Akaike weights, while taking the number of model comparisons into account, do not assess the quality of the model in an absolute sense. There may well be other models, which we did not explore, that would give yet better accounts of our data, even though the fits shown in Fig.7 and the variance accounted for (as reported) demonstrate that the models did capture the features of the data well. Our results demonstrate that there are conditions (and areas) where a single attention mechanism is unable to account for the effects seen in all neurons. This finding emphasizes the importance of exploring different models (in conjunction with model validation) to understand brain mechanisms that support cognitive operations.

The discrepancy between our data and previously published data (Ni *et al*., 2012) begs the question of where the differences arise from. Are they due to differences in the areas recorded from, for example V1 being an early visual cortical area which shows smaller attention effects than MT? Do they arise from differences in experimental design, i.e. attention being directed to the RF and away from it (our study) vs. towards two different locations/stimuli inside the RF (Ni *et al*., 2012)? Would our results change if attention was directed to different locations within the same visual hemifield? Or do the differences arise from differences in visual stimulation, i.e. single vs. multiple stimuli inside the RF, and normalization inside (two stimuli inside the RF) vs. outside the RF (surround stimulus presentation)? At this stage we can only speculate, but it is possible that area differences contribute to the discrepancy. In area V1 increased normalization (by increasing the stimulus size or increasing the contrast) results not only in altered spiking activity but also in increased oscillatory activity in the gamma frequency range (Henrie & Shapley, 2005; Gieselmann & Thiele, 2008; Ray & Maunsell, 2010). At the same time, attention (to a very small part of visual space) in primary visual cortex reduces oscillatory activity in the gamma frequency band (Chalk *et al*., 2010; Herrero *et al*., 2013), i.e. it has opposite effects to those seen by increasing normalization. Thus, attention may serve as a surround exclusion mechanism (Roberts *et al*., 2007; Sundberg *et al*., 2009), whereby it reduces the normalization induced by surround stimuli. At the same time, differences in experimental design could also account for the differences found. In Ni *et al*.'s study attention was directed either to a preferred or an anti-preferred stimulus inside the RF (they also had an outside attend control condition), while in our study attention was either directed to the RF centre (a 0.1*0.1° patch representing the behaviourally relevant location), or into the opposite hemifield. Shifting attention between different parts of the RF (Ni *et al*., 2012) may engage different (normalization) mechanisms than shifting attention between the RF centre and a location in the opposite hemifield (our study). Moreover, in our study the stimuli inducing normalization were presented largely outside the classical RF, while they occupied parts of the RF centre in Ni *et al*.'s (2012) study. Given that normalization models argue that attention affects neuronal processing by interacting multiplicatively with the stimulus drive *and* with the suppressive drive (Reynolds & Heeger, 2009), while Cavanaugh *et al*. (2002a) suggest that centre and surround gains are regulated independently in V1 (i.e. not by a single source), it is conceivable that the task/stimulus differences outlined above are critical in terms of whether attention and normalization affect the same circuitry. Designing a study similar to Ni *et al*. (2012) for area V1 is very difficult, if not impossible (due to their small size of V1 parafoveal RFs). However, our study design could be applied to MT (or V4). This would answer whether the discrepancies are based on area or design differences or both. A partial answer to that question can already be obtained from reports for area V4 (Ghose & Maunsell, 2008), where the effects of attention were measured under conditions similar to those reported by Ni *et al*. (2012). Ghose & Maunsell (2008) reported that attention affects mostly the input gain of V4 neurons, which thereby allows neurons to differently weigh relevant vs. irrelevant incoming information. Their study did not find strong evidence for normalization to contribute to attentional modulation, even though for some neurons (∽25–30% depending on the model tested) the fits were significantly improved when a free parameter of power (equivalent to some form of normalization) was added to the model. This proportion of neurons is roughly equivalent to what we found. Based on these multiple findings we suggest that attention affects neuronal responses by multiple mechanisms, whereby the relative weighting of these mechanisms may be affected by the stimulus and task conditions, and possibly also by the architecture of the area where responses are measured. Neurons that were best fitted with normalizing attention vs. non-normalizing attention models possibly have different connections with downstream areas and also may receive different feedback signals. The increase in firing rate usually seen with attention could be implemented by indirect feedback connection from parietal or frontal areas (via extrastriate areas), targeting excitatory and inhibitory neurons in V1. Given our results of non-normalizing models being the better descriptors for the majority of cells, the latter may not be very prominent. However, an increase in neuromodulator tone such as acetylcholine could also result in increased firing rates (Herrero *et al*., 2008), either by directly affecting firing rates on a trial by trial basis, or by allowing feedback to have increased impact (Deco & Thiele, 2011). These hypotheses can probably soon be tested with the advancement in optogenetics in primates. Whatever the answer to these questions, our current results demonstrate that there are conditions where attention does not contribute to normalization in the majority of V1 neurons.

## Abbreviations

AIC: Akaike's information criterion
BIC: Bayesian information criterion
MI: modulation index
MT: middle temporal area
PSTH: peri-stimulus–time histogram
RF: receptive field
SI: suppression index
